# Distinct Temporal Expression of 5-HT_1A_ and 5-HT_2A_ Receptors on Cerebellar Granule Cells in Mice

**DOI:** 10.1007/s12311-014-0565-4

**Published:** 2014-05-01

**Authors:** Marlies Oostland, M. Renate Buijink, Guus M. Teunisse, Lars von Oerthel, Marten P. Smidt, Johannes A. van Hooft

**Affiliations:** Swammerdam Institute for Life Sciences, Center for Neuroscience, University of Amsterdam, P.O. box 94232, 1090 GE Amsterdam, The Netherlands

**Keywords:** Serotonin receptors, Cerebellum, Synaptic plasticity

## Abstract

Serotonin plays an important role of controlling the physiology of the cerebellum. However, serotonin receptor expression has not been fully studied in the developing cerebellum. We have recently shown that cerebellar granule cells transiently express 5-HT_3_ receptors. In the present study, we investigate expression of 5-HT_1_ and 5-HT_2_ receptors in the mouse cerebellum both during postnatal development and in juvenile mice. Here, we show for the first time that 5-HT_1A_ and 5-HT_2A_ receptors are present on cerebellar granule cells with a distinct temporal expression pattern: 5-HT_1A_ receptors are expressed only during the first 2 weeks, while 5-HT_2A_ receptor expression persists until at least 8 weeks after birth. Because of its prolonged expression pattern, we investigated the electrophysiological properties of the 5-HT_2A_ receptor. 5-HT_2A_ receptors expressed by cerebellar granule cells promote stability by reducing variability of the synaptic response, and they modulate the paired-pulse ratio of the parallel fibre–Purkinje cell synapse. Furthermore, pharmacological block of 5-HT_2A_ receptors enhances short-term synaptic plasticity at the parallel fibre–Purkinje cell synapse. We thus show a novel role for serotonin in controlling function of the cerebellum via 5-HT_2A_ receptors expressed by cerebellar granule cells.

## Introduction

The cerebellum does not only play an important role in motor coordination and motor learning, but is also involved in cognitive processes. Several studies have discussed the involvement of the cerebellum in neurodevelopmental disorders such as autism [1–3] and schizophrenia [4–7]. These neurodevelopmental disorders are associated with a change in serotonin receptor expression in the cerebellum [8, 9]. It is known that the cerebellum of the rodent brain receives innervation of serotonergic fibres, originating mainly from the medullary and pontine reticular formation [10, 11]. These serotonergic fibres are predominantly found around the somata of Purkinje cells and in the overlying molecular layer, which contains the dendrites of Purkinje cells. However, during development of the cerebellum, the serotonergic system and its physiological significance are not fully understood.

We have recently shown that 5-HT_3_ receptors are transiently expressed by cerebellar granule cells in mice during early postnatal development [12]. Pharmacological block of 5-HT_3_ receptors impairs synaptic plasticity at glutamatergic inputs targeting Purkinje cells during this developmental period [12]. Furthermore, 5-HT_3_ receptors regulate the morphological maturation of Purkinje cells [13]. 5-HT_3A_ receptor knockout mice show delayed climbing fibre elimination due to impaired plasticity at the parallel fibre–Purkinje cell synapse [13]. We hypothesized that other members of the serotonergic system in the cerebellum co-regulate cerebellar development and are functional at time points surrounding the transient expression pattern of 5-HT_3_ receptors. This happens presumably by switching to other types of serotonin receptors expressed in the cerebellum during postnatal development and thereafter. Studies using autoradiography and immunohistochemistry have shown that multiple different 5-HT receptor subtypes are present on Purkinje cells, including the 5-HT_1A_, 5-HT_2A_, 5-HT_2B_, 5-HT_2C_, 5-HT_5A_ and 5-HT_7_ subtypes [14–19]. On cerebellar granule cells, the expression of 5-HT receptors is less diverse. It has been reported that both 5-HT_3_ and 5-HT_6_ receptors are present on these cells [12, 18, 20, 21]. In addition, very low densities of 5-HT_1_ receptors in the molecular and granule cell layer in the adult rat cerebellum have been found [22]. There is some evidence suggesting presence of 5-HT_2_ receptors on dissociated cerebellar granule cells at P8 from rat cerebellum [23, 24]. Other studies done on adult rodents did not reveal the presence of 5-HT_2_ receptors on cerebellar granule cells [18, 25].

The aim of the present study is to investigate the expression pattern and functional properties of 5-HT_1A_ and 5-HT_2A_ receptors in the mouse cerebellum. We show for the first time that functional 5-HT_1A_ and 5-HT_2A_ receptors are expressed by cerebellar granule cells during early postnatal development. 5-HT_1A_ receptors are transiently expressed during the first 2 weeks postnatally, while 5-HT_2A_ receptors remain present on granule cells until 10 weeks of age. We furthermore investigate the electrophysiological properties of 5-HT_2A_ receptors in the cerebellum and conclude that they modulate plasticity at the parallel fibre–Purkinje cell synapse.

## Materials and Methods

### Ethical Approval

Wild-type C57/Bl6 mice (Harlan, www.harlan.com) between the age of postnatal day (P) 2 and P90, both males and females, were used for this study. All experiments in this study were performed in accordance with the committee on animal bioethics of the University of Amsterdam, which specifically approved this study. All efforts were made to minimize suffering.

### Immunohistochemistry

Brains of wild-type C57/Bl6 mice at P5 and P90 where isolated in ice-cold phosphate-buffered saline (PBS) and fixated in 4 % PFA in PBS for 6 h. Sagittal (P5) or coronal (P90) sections were cut from the cerebellum at 16 μm and mounted on slides. Slides were washed for 3 × 5 min in PBS, and blocking was performed in 4 % heat-inactivated foetal calf serum in PBS-0.25 % Triton (PBS-T). Primary antibody against the 5-HT_2A_ receptor (Cat no: 24288, Immunostar) was incubated at a dilution of 1:500 in PBS-T for 1 h at room temperature and 4 °C overnight. Excessive antibody was removed by washing 3 × 5 min in PBS, and secondary antibody (goat anti-rabbit Alexa 488, A11008 Molecular Probes) was incubated at a dilution of 1:1,000 in PBS-T for 2 h. Slides were washed for 2 × 5 min in PBS and a 4′,6-diamidino-2-phenylindole (DAPI) staining was performed for 5 min (1:3,000 dilution of staining solution in PBS, original staining solution stock 1 mg/ml, D9564 Sigma, in H_2_O). Slides were washed 2 × 10 min in PBS and embedded with FluorSave (345789-20 Calbiochem). Fluorescent pictures were taken using a Zeiss fluorescence microscope and MetaMorph acquisition software.

### Electrophysiological Recordings

For whole-cell patch-clamp recordings, animals were killed by decapitation. Sagittal brain slices were cut using a vibrating blade microtome (Leica VT1200S) at a thickness of 300 μm. During slicing, the brains were kept in cooled (4 °C) oxygenated artificial cerebrospinal fluid (ACSF) which was composed of the following (in mM): NaCl (120), KCl (3.5), CaCl_2_ (2.5), MgSO_4_ (1.3), NaH_2_PO_4_ (1.25), NaHCO_3_ (25) and d-glucose (25), continuously bubbled with 95 % O_2_ and 5 % CO_2_ (pH = 7.4). Brains from mice older than 3 weeks were sliced in modified ACSF, composed of the following (in mM): choline chloride (120), KCl (3.5), CaCl_2_ (0.5), MgSO_4_ (6.0), NaH_2_PO_4_ (1.25), d-glucose (25) and NaHCO_3_ (25). During the experiments, slices were kept submerged at room temperature and continuously superfused with ACSF. Patch pipettes were pulled from borosilicate glass with a resistance of 2–3 MΩ for recordings from Purkinje cells and with a resistance of 8–10 MΩ for recordings from granule cells. Patch pipettes were filled with internal solution containing the following (in mM): K gluconate (110), KCl (30), EGTA (0.5), HEPES (10), Mg-ATP (4) and Na-GTP (0.5; pH 7.3 with KOH). Whole-cell recordings were made at room temperature using an EPC9 patch-clamp amplifier and PULSE software (HEKA Electronic, Lambrecht, Germany). Signals were filtered at 1–5 kHz and sampled at 10 kHz. Series resistance ranged from 2 to 11 MΩ and was compensated for at least 60 %. All recordings are corrected for liquid junction potential. Cells were voltage clamped at −70 mV.

Whole-cell patch-clamp recordings were made from granule cells (at P2–P68) with a second pipette connected to a picospritzer II (General Valve, Fairfield, NJ, USA) containing 100 nM of the 5-HT_1A_ agonist 8-hydroxy-2-(di-n-propylamino)tetralin (8-OH-DPAT; gift from Abbott Laboratories BV), 1 μM of the specific 5-HT_2B/C_ agonist (S)-6-chloro-5-fluoro-1H-indole-2-propanamine (RO60-0175, gift from Abbott Laboratories BV), or 500 nM of the 5-HT_2_ agonist 2,5-dimethoxy-4-iodoamphetamine (DOI, gift from Abbott Laboratories BV) in ACSF was positioned in the vicinity of the cell soma, and the drug was applied for 500 ms at 35–100 kPa. Antagonists were applied in the bath solution, and slices were preincubated in the antagonist for >30 min. We used 10 μM *N*-[2-[4-(2-methoxyphenyl)-1-piperazinyl]ethyl]-*N*-(2-pyridyl)cyclohexanecarboxamide (WAY 100,635, gift from Abbott Laboratories BV) to block 5-HT_1A_ receptors and 300 nM (R)-(+)-α-(2,3-dimethoxyphenyl)-1-[2-(4-fluorophenyl)ethyl]-4-piperidinemethanol (MDL 100,907, gift from Abbott Laboratories BV) to block 5-HT_2A_ receptors.

Miniature postsynaptic currents from Purkinje cells were recorded in the voltage-clamp configuration in the presence of 0.5 μM TTX (Latoxan, Valence, France) and analyzed as described before [26]. Miniature excitatory postsynaptic currents (mEPSCs) were recorded with additional 20 μM bicuculline (Tocris Bioscience) in the bath solution, while miniature inhibitory postsynaptic currents (mIPSCs) were recorded with 20 μM 6-cyano-7-nitroquinoxaline-2,3-dione (CNQX; Tocris Bioscience) in the bath solution. Per cell, one or two traces of 5 min each were used for analysis, with at least 100 miniature synaptic events per cell which were visually verified. A cumulative distribution was made for each cell, and all distributions were averaged to get the final cumulative distribution as shown in Fig. 3. In this way, all cells contribute equally to the final distribution.

Glutamatergic synaptic currents in Purkinje cells were evoked by stimulation of the parallel fibres with a glass electrode filled with ACSF. Paired stimuli (100–400 μA, 0.2 ms duration, interstimulus interval 50 ms) were delivered to the internal granule cell layer using a custom-made isolated bipolar current stimulator. Paired-pulse stimulations were delivered with a 20-s interval, and only recordings which were stable for at least 15 min were used to analyze the paired-pulse ratio (PPR). Recordings used for analysis were checked to have a gradual increase in amplitude upon a gradual increase in stimulus intensity and paired-pulse facilitation, both features of the parallel fibre–Purkinje cell synapse. The PPR was defined as the amplitude of the second EPSC divided by the amplitude of the first EPSC. The coefficient of variance (CV) was determined for cells that had a stable baseline for at least 50 stimuli in each condition. The CV was calculated for each cell by dividing the standard deviation by the mean of the EPSC amplitude.

For synaptic plasticity at the parallel fibre–Purkinje cell synapse, recordings were made from Purkinje cells as described above. Synaptic plasticity was induced using a low frequency stimulation protocol with a tetanus current injection of 8 Hz for 30 s [27], at a stimulus intensity which gave a postsynaptic current below the maximum induced postsynaptic current. The area of the EPSCs was used to analyze the postsynaptic responses. To investigate the time course of the synaptic plasticity onset, from some cells we recorded EPSCs throughout the complete time course (see example in Fig. 4c). In all cells, we recorded EPSCs at least during the 10–15 min after induction of plasticity. The analysis of these EPSCs is shown in Fig. 4d.

### Statistical Analysis

Values are expressed as mean ± standard error of the mean. Comparisons were made using the Student *t* test unless stated otherwise. A *p* < 0.05 was used to indicate a significant difference. Asterisks indicate *p* < 0.05 (*), *p* < 0.01 (**) and *p* < 0.001 (***).

## Results

### Cerebellar Granule Cells Express 5-HT_1A_ and 5-HT_2A_ Receptors During Postnatal Development

**Fig. 1:**
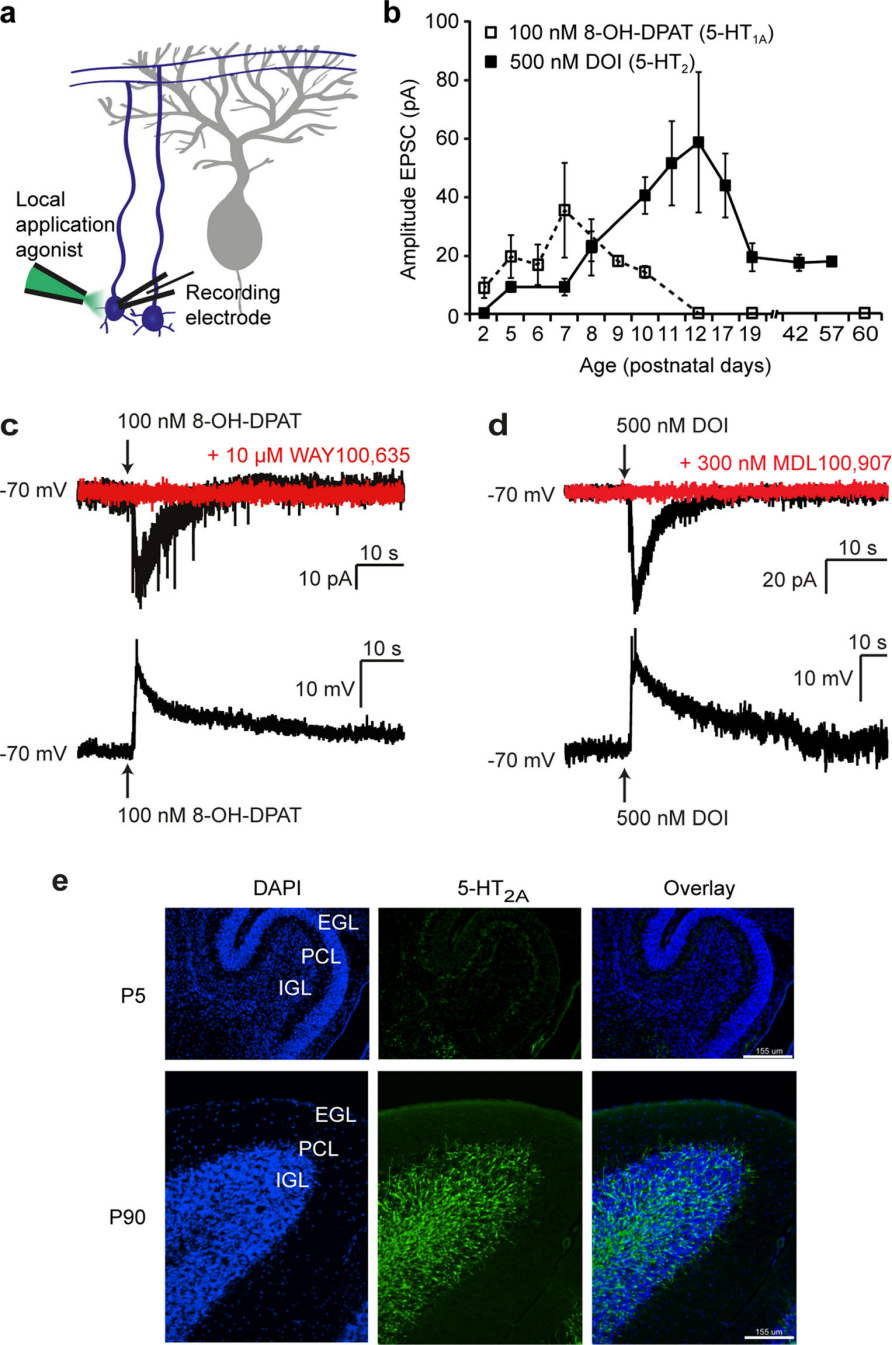
Cerebellar granule cells express functional 5-HT_1A_ and 5-HT_2A_ receptors during postnatal development. **a** Schematic diagram of the electrophysiological recording configuration. **b** Amplitudes of inward currents recorded from granule cells, evoked by local application of 100 nM 8-OH-DPAT or 500 nM DOI at different ages during postnatal development. **c** Examples of a whole-cell patch-clamp recording from a granule cell in voltage-clamp (*top*) and current-clamp (*bottom*) at P8 showing an inward current and a depolarization of the membrane potential upon local application of 100 nM of the 5-HT_1A_ agonist 8-OH-DPAT. Also shown in *red* is an example trace of a recording in voltage-clamp after preincubation of the slice in 10 μM of the 5-HT_1A_ specific antagonist WAY100,635, which blocks the effect of local application of 100 nM 8-OH-DPAT. **d** Examples of a whole-cell patch-clamp recording from a granule cell in voltage-clamp (*top*) and current-clamp (*bottom*) at P11, showing an inward current and depolarization of the membrane potential upon local application of 500 nM of the 5-HT_2_ specific agonist DOI. Also shown in *red* is an example trace of a recoding in voltage-clamp after preincubation of the slice in 300 nM MDL 100,907, indicating no inward current upon local application of 500 nM DOI. *Arrows* in **b** and **c** indicate the start of drug application which lasts for 500 ms. **e** Immunostaining with DAPI and antibodies against 5-HT_2A_ receptors (obtained from Immunostar) at P5 and at P90, indicating presence of 5-HT_2A_ receptors both on Purkinje cells and in the granule cell layer, with higher expression levels in adult mice. *EGL* external granule cell layer, *PCL* Purkinje cell layer, *IGL* internal granule cell layer. *Scale bar* indicates 155 μm

The expression of 5-HT_1A_ and 5-HT_2A_ receptors on cerebellar granule cells during postnatal development was studied using whole-cell patch-clamp recordings and local application of selective agonists (Fig. 1a). A low concentration (100 nM) of the 5-HT_1A_ agonist 8-OH-DPAT was used to prevent activation of the 5-HT_7_ receptors, for which 8-OH-DPAT has a low affinity [28]. Figure 1b shows that functional 5-HT_1A_ receptors are expressed by cerebellar granule cells during the first 10 days postnatally but not at later ages. Local application of 100 nM 8-OH-DPAT to granule cells at P6–P9 resulted in an inward current of 19.7 ± 2.2 pA (*n* = 11 cells, 4 mice, example trace in Fig. 1c, top) in voltage-clamp, and in a depolarization of 17.6 ± 3.5 mV (*n* = 5, example trace in Fig. 1c, bottom) in current-clamp. The 5-HT_1A_ receptor-mediated inward current in granule cells could be blocked in 6 out of 7 cells (3 mice) by bath application of 10 μM of the 5-HT_1A_ specific antagonist WAY100,635 (Fig. 1c).

Conversely, 5-HT_2A_ receptors are expressed by cerebellar granule cells from 5 days after birth, with a peak in expression levels around the end of the second postnatal week and with lower but sustained expression at later ages until at least 10 weeks old. Local application of 500 nM of the 5-HT_2_ agonist DOI to cerebellar granule cells resulted in an inward current of 49.7 ± 8.8 pA (*n* = 19 cells, 5 mice, example in Fig. 1c, top) at P10–P15 in voltage-clamp configuration and in a depolarization of 12.8 ± 2.7 mV (*n* = 6, example in Fig. 1c, bottom) in current-clamp configuration. As DOI cannot discriminate between the different 5-HT_2_ receptor subtypes, 1 μM RO60-0175, a specific 5-HT_2B/C_ agonist, was locally applied to the cerebellar granule cells. In contrast to DOI, RO60-0175 did not evoke an ion current at P10 (*n* = 6; data not shown). Application of 300 nM MDL 100,907, a 5-HT_2A_ receptor-specific antagonist, blocked the DOI-induced ion current at P11 (*n* = 3 cells, 3 mice, example in Fig. 1c, top) and at P42 (*n* = 3 cells, 3 mice). The expression pattern of the 5-HT_2A_ receptor was confirmed by immunohistochemistry using a specific 5-HT_2A_ receptor antibody (Fig. 1e). Because of the temporal restriction of 5-HT_1A_ receptors on cerebellar granule cells to the first 10 days postnatally and because of the sustained expression of 5-HT_2A_ receptors, the functional role of 5-HT_2A_ receptors on granule cells was studied in more detail.

### Presynaptic 5-HT_2A_ Receptors Modulate Paired-Pulse Ratio and Stability at the Parallel Fibre–Purkinje Cell Synapse

**Fig. 2:**
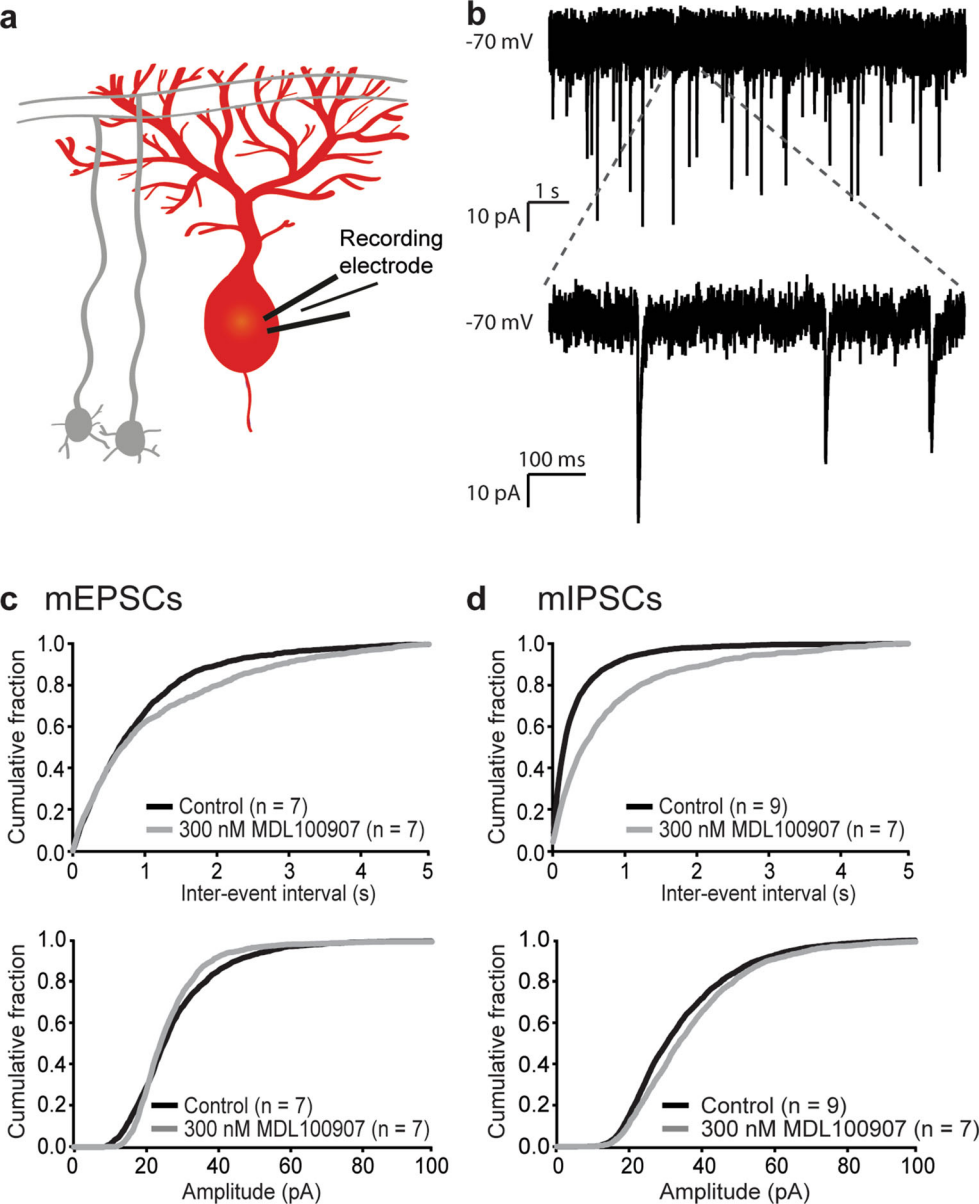
Presynaptic 5-HT_2A_ receptors modulate the frequency of spontaneous mEPSCs and mIPSCs recorded from Purkinje cells. **a** Schematic diagram of the electrophysiological recording configuration of **b**–**d. b** Example trace (with magnification) of miniature inhibitory synaptic currents recorded from Purkinje cells at −70 mV. **c** Inter-event interval distributions and amplitude distributions of mEPSCs recorded from Purkinje cells show an increase in inter-event interval (a reduction in frequency) and a difference in mEPSC amplitude after application of 300 nM of the selective 5-HT_2A_ receptor antagonist MDL 100,907. **d** Inter-event interval distributions and amplitude distributions of mIPSCs recorded from Purkinje cells show an increase in inter-event interval (a reduction in frequency) but no difference in mIPSC amplitude after application of 300 nM of the selective 5-HT_2A_ receptor antagonist MDL 100,907

To further investigate the presence of 5-HT_2A_ receptors presynaptically on excitatory synapses onto Purkinje cells, miniature postsynaptic spontaneous events were recorded from Purkinje cells (Fig. 2a, with example trace in Fig. 2b) from control slices and from slices which were preincubated in 300 nM of the 5-HT_2A_ receptor antagonist MDL 100,907. Miniature excitatory postsynaptic currents (mEPSCs) were recorded in the presence of 0.5 μM TTX and 20 μM bicuculline. Both inter-event interval and amplitude distributions showed a difference in the presence of MDL 100,907 (both *p* < 0.01, Kolmogorov–Smirnov test, Fig. 2c). This confirms our earlier findings that 5-HT_2A_ receptors are located postsynaptically on Purkinje cells, and furthermore indicates presence of 5-HT_2A_ receptors presynaptically on excitatory synapses at Purkinje cells. Miniature inhibitory postsynaptic currents (mIPSCs) were recorded in the presence of 0.5 μM TTX and 20 μM CNQX. The inter-event interval distributions of mIPSCs recorded from Purkinje cells in control slices or slices preincubated with MDL 100,907 were different (*p* < 0.001, Kolmogorov–Smirnov test, Fig. 2d, top). This shows that 5-HT_2A_ receptors are presynaptically located at synapses of inhibitory inputs onto Purkinje cells. There was no difference in amplitude distributions of the mIPSCs, suggesting that 5-HT_2A_ receptors on Purkinje cells do not modulate postsynaptic GABA receptors (Fig. 2d, bottom).

**Fig. 3:**
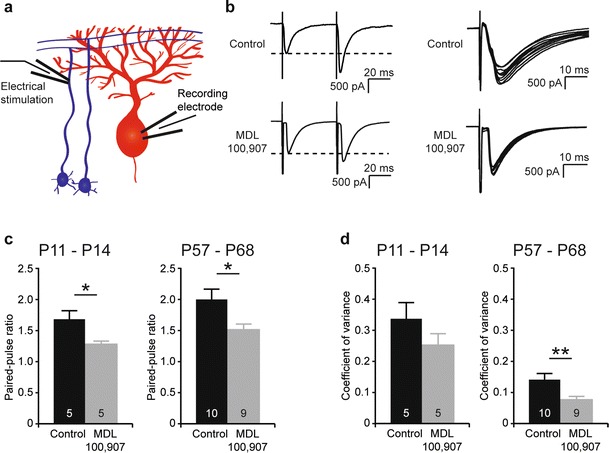
Presynaptic 5-HT_2A_ receptors modulate stability of the parallel fibre–Purkinje cell synapse. **a** Schematic diagram of the electrophysiological recording configuration of **b**–**d. b** Examples of currents recorded from Purkinje cells at P11–P14 after electrically stimulating parallel fibres with a double-pulse protocol, and examples of ten consecutive currents recorded from Purkinje cells at P11–P14 after electrically stimulating parallel fibres, both in control slices and in slices preincubated with 300 nM MDL 100,907. **c** Pharmacological blockade of 5-HT_2A_ receptors with 300 nM MDL 100,907 results in a reduced paired-pulse ratio at both P11–P14 and at P57–P68. **d** At P11–P14, the effect of pharmacological blockade of 5-HT_2A_ receptors with 300 nM MDL 100,907 on the coefficient of variance of EPSCs at the parallel fibre–Purkinje cell synapse does not reach significance. At P57–P68, blocking 5-HT_2A_ receptors results in a lower coefficient of variance of EPSCs at the parallel fibre–Purkinje cell synapse. The *numbers* in the bars of the graphs indicate the number of cells used for analysis

Presynaptic 5-HT_2A_ receptors present on cerebellar granule cells were studied for their role in the short-term plasticity of the parallel fibre–Purkinje cell synapse. Whole-cell patch-clamp recordings were made from Purkinje cells while electrically stimulating parallel fibres in the internal granule cell layer (Fig. 3a, b). At P11–P14, short-term synaptic plasticity at the parallel fibre–Purkinje cell synapse was lower after pharmacological block of 5-HT_2A_ receptors. The average paired-pulse ratio was 1.67 ± 0.14 in control slices (*n* = 5 cells, 3 mice) and 1.27 ± 0.04 in the presence of 300 nM MDL 100,907 (*n* = 5 cells, 3 mice, *p* < 0.05, Fig. 3c). The effect of 5-HT_2A_ receptors on short-term plasticity at the parallel fibre–Purkinje cell synapse was also tested in 8- to 10-week-old mice (P57–P68). Here, the paired-pulse ratio was also lower after pharmacological block of 5-HT_2A_ receptors, from 1.98 ± 0.17 in control slices (*n* = 10 cells, 5 mice) to 1.50 ± 0.08 in the presence of 300 nM MDL 100,907 (*n* = 9 cells, 5 mice, *p* < 0.05, Fig. 3c).

The stability of the EPSC at the parallel fibre–Purkinje cell synapse was investigated using the same electrophysiological recording configuration as described above (Fig. 3a, b). At P11–P14, the effect of a pharmacological block of 5-HT_2A_ receptors on synapse stability does not reach statistical significance, with a coefficient of variance of 0.33 ± 0.05 in control slices (*n* = 5) and of 0.25 ± 0.04 after blocking 5-HT_2A_ receptors with MDL 100,907 (*n* = 5, Fig. 3d). However, in 8- to 10-week-old mice (P57–P68), recordings from Purkinje cells in control slices had a higher variability of EPSC amplitude. The coefficient of variance had an average of 0.14 ± 0.02 in control slices compared to 0.076 ± 0.009 in slices preincubated in MDL 100,907 (*n* = 9, *p* < 0.001, Fig. 3d).

### Block of 5-HT_2A_ Receptors Enhances Synaptic Plasticity

**Fig. 4:**
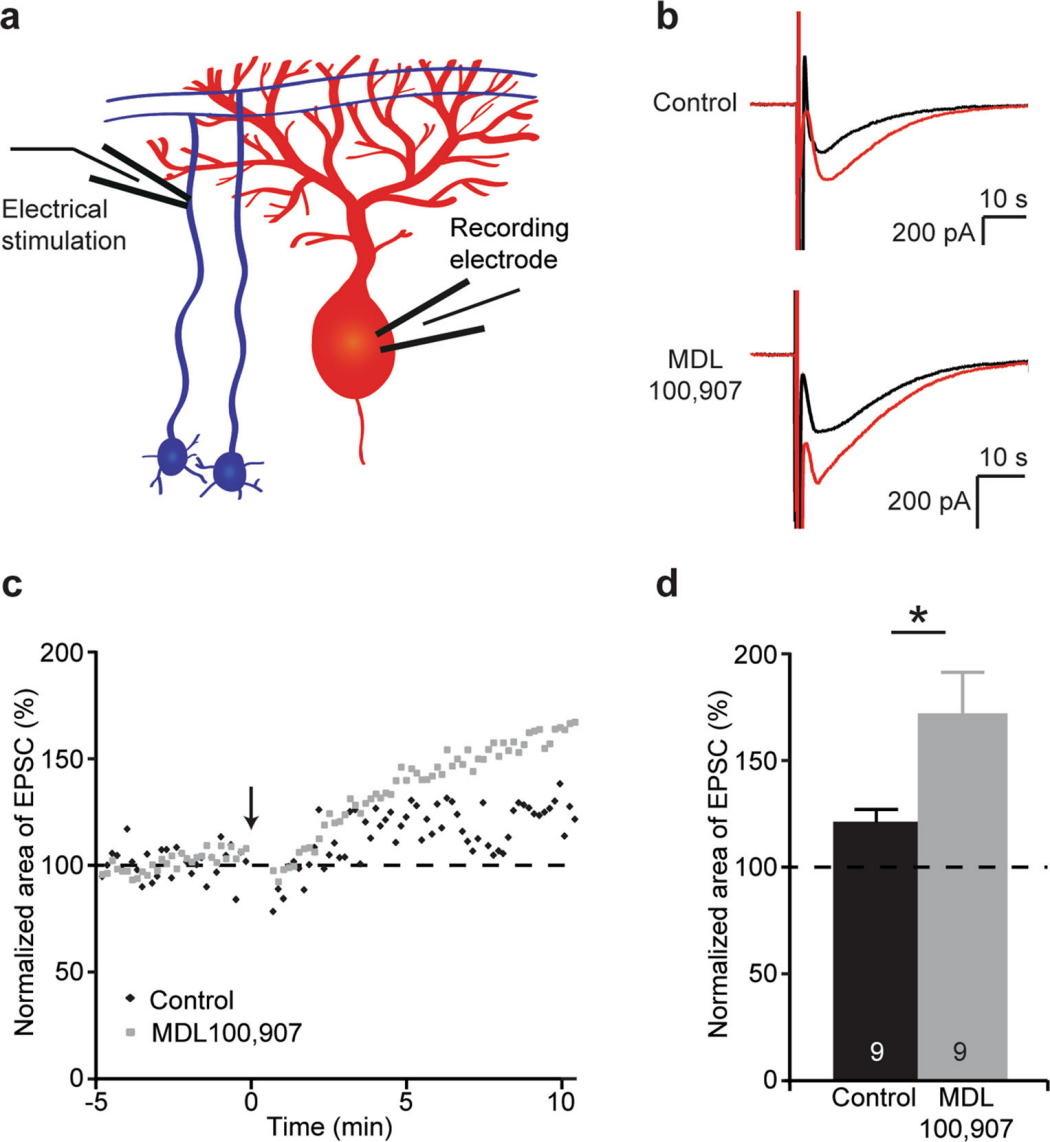
5-HT_2A_ receptors modulate synaptic plasticity at the parallel fibre–Purkinje cell synapse in 8- to 10-week-old mice. **a** Schematic diagram of the electrophysiological recording configuration of **b**–**d. b** Examples of recordings from Purkinje cells while electrically stimulating the parallel fibres with a double-pulse protocol, from a control slice and from a slice which is preincubated with 300 nM of the 5-HT_2A_ selective antagonist MDL 100,907. *Black traces* are from recordings before inducing plasticity; *red traces* are from recordings 10–15 min after induction of plasticity using a 8 Hz, 30 s stimulation protocol. **c** Example of recordings from Purkinje cells from a control slice and from a slice which is preincubated with 300 nM of the 5-HT_2A_ selective antagonist MDL 100,907 in which the area of the EPSC, normalized to the average before the tetanus protocol, is recorded over time. The *arrow* indicates the start of the tetanus to induce plasticity with a 8 Hz, 30 s stimulation protocol. **d** The area of EPSCs recorded 10–15 min after the plasticity stimulation protocol, normalized to the area of EPSCs before the plasticity stimulation protocol, indicates enhanced plasticity after blocking 5-HT_2A_ receptors with MDL 100,907. The *numbers* in the bars of the graphs indicate the number of cells used for analysis

Given that 5-HT_2A_ receptors are expressed presynaptically and modulate the stability of synaptic transmission, we further investigated the role of the 5-HT_2A_ receptors in synaptic plasticity. As pharmacologically blocking 5-HT_2A_ receptors increased the stability of the EPSC at the parallel fibre–Purkinje cell synapse, we hypothesized that functional 5-HT_2A_ receptors impair presynaptic synaptic plasticity at the parallel fibre–Purkinje cell synapse. To test this, whole-cell patch-clamp recordings were made from Purkinje cells from 8- to 10-week-old mice while electrically stimulating the parallel fibres in the internal granule cell layer with a tetanus of 8 Hz during 30 s (Fig. 4a) in the absence and presence of 300 nM MDL 100,907 (Fig. 4b). Indeed, synaptic plasticity was increased after pharmacological block of 5-HT_2A_ receptors. The difference between control and MDL 100,907-treated slices became evident from 5 min after the tetanus protocol (example in Fig. 4c). A higher increase of the area of the EPSC 10–15 min after induction of plasticity by the tetanus protocol was found in slices treated with MDL 100,907 (Fig. 4d). Thus, we conclude that blocking 5-HT_2A_ receptors enhances short-term synaptic plasticity in 8- to 10-week-old mice.

## Discussion

In this study, we show a novel way for serotonin to control development of the cerebellum, mediated via 5-HT_2A_ receptors present on cerebellar granule cells during postnatal development and until at least 8 weeks after birth. We show the electrophysiological effects of 5-HT_1A_ and 5-HT_2A_ receptors present on granule cells during postnatal development of the cerebellum. We further characterize 5-HT_2A_ receptor-mediated currents and conclude that 5-HT_2A_ receptors expressed by granule cells are involved in mediating stability and short-term synaptic plasticity at the parallel fibre–Purkinje cell synapse.

### Expression Pattern of 5-HT_1A_ and 5-HT_2A_ Receptors in the Cerebellum

We show for the first time expression of 5-HT_1A_ and 5-HT_2A_ receptors by cerebellar granule cells. The expression patterns of both 5-HT_1A_ and 5-HT_2_ receptors on cerebellar granule cells mimic the known expression pattern of these receptors on Purkinje cells. Activation of these receptors leads to an inward current in voltage-clamp configuration and to a depolarization in current-clamp configuration.

Electrophysiological evidence indicates that expression of 5-HT_1A_ receptors by cerebellar granule cells peaks at P7, after which expression decreases, and no functional 5-HT_1A_ receptors are present from P12 onward. In Purkinje cells, 5-HT_1A_ receptor expression occurs during the first week postnatally, decreases thereafter, and no expression is detected in adult rodents [14, 29–31]. This makes the 5-HT_1A_ receptor the earliest serotonin receptor to be expressed in the cerebellum during postnatal development, and the expression pattern coincides with the expression pattern of serotonergic fibres in the cerebellar cortex. In rodents, serotonergic fibres appear in the white matter around birth. During the first postnatal week, serotonergic fibres extend into the internal granule cell layer, occasionally also penetrating the Purkinje cell layer. The serotonergic fibres extend further into the cerebellar cortex during the second and third postnatal week [32]. The first serotonergic effects in the cerebellum after birth are therefore likely to be mediated via 5-HT_1A_ receptors on Purkinje cells and granule cells. Their functional role at such an early age, after which they disappear, remains to be further investigated.

Functional 5-HT_2A_ receptors are not present on cerebellar granule cells before P5, after which their expression increases until it reaches a peak around 2 weeks after birth. Sustained expression of functional 5-HT_2_ receptors on cerebellar granule cells is found until 8 weeks after birth. This also mimics 5-HT_2_ receptor expression by Purkinje cells, which begins around P0–P5 and gradually increases until P21, as shown by immunohistochemistry [19, 33]. The effects of 5-HT_2_ receptors during the postnatal development of the cerebellum were examined by recording spontaneous miniature events from Purkinje cells. Pharmacological block of 5-HT_2A_ receptors resulted in an increase in inter-event interval and a decrease in amplitude of the mEPSCs. These data confirm that the 5-HT_2A_ receptor is located both on Purkinje cells and on excitatory presynaptic inputs to the Purkinje cells. We conclude that the excitatory presynaptic cells expressing 5-HT_2A_ receptors are the granule cells, which we have shown to express 5-HT_2A_ receptors using immunohistochemistry and electrophysiological experiments described above. Furthermore, an increase in inter-event interval of the mIPSCs was found after blocking 5-HT_2A_ receptors, indicating presence of 5-HT_2A_ receptors on presynaptic inhibitory inputs to the Purkinje cells. Of the inhibitory cells in the cerebellum, the Golgi cell is the only cell type known to express the 5-HT_2A_ receptor; however, these cells have no synapses on Purkinje cells [18]. Inhibitory basket and stellate cells do synapse on the Purkinje cell, but these cells are not known to be 5-HT sensitive [34]. A possible candidate is the Lugaro cell, which is sensitive to 5-HT and has 5-HT driven inhibitory synapses on Purkinje cells [34, 35].

### Concluding Remarks

We have shown presence and functional consequences of 5-HT_1A_ and 5-HT_2A_ receptors on cerebellar granule cells in this study and recently also presence and functional consequences of 5-HT_3_ receptors on cerebellar granule cells [12, 13]. All three serotonin receptors are expressed during the first 3 weeks after birth, but their specific temporal expression pattern is different [36]. 5-HT_1A_ receptors are expressed earliest during the first postnatal week, and both 5-HT_2A_ and 5-HT_3_ receptors are expressed during the second postnatal week, but only 5-HT_2A_ receptor expression remains until at least postnatal week 10. It has been shown before that functional interplays between serotonin receptors occur. Activation of 5-HT_2_ receptors can potentiate 5-HT_3_ receptor function in rat trigeminal ganglion neurons via involvement of G proteins and PKC [37]. 5-HT_2_ and 5-HT_3_ receptors have a similar rise of expression during the first postnatal week in rodents, but while 5-HT_3_ receptor expression by cerebellar granule cells decreases after 2 weeks with no 5-HT_3_ receptors left at 3 weeks postnatally, 5-HT_2_ receptors persist to be expressed by granule cells until at least 8 weeks postnatally. We assume that during the first 3 weeks, 5-HT_2_ and 5-HT_3_ receptors strengthen each other’s function in postnatal development of the cerebellum, with 5-HT_2_ receptors persisting in this function when 5-HT_3_ receptors gradually disappear. Functional 5-HT_2_ receptors reduce strength of synaptic activity and reduce stability at the parallel fibre–Purkinje cell synapse. Activity-dependent strengthening of the synapse could affect maturation, with 5-HT_2_ receptor-mediated activity slowing down the maturational process.

Taken together, these results show that serotonin is in a powerful position to control the physiology of the cerebellum at all ages, ranging from newborn to adult, through distinct temporal expression of its receptors, not only on Purkinje cells but also on granule cells.
